# In-Hospital Outcomes of Inflammatory Bowel Diseases in Patients With Diabetes Mellitus: A Propensity Score Matching Analysis

**DOI:** 10.7759/cureus.16566

**Published:** 2021-07-22

**Authors:** Ese Uwagbale, Omolara G Adeniran, Olayemi A Adeniran, Ifeanyichukwu Onukogu, Solomon Agbroko, Niket Sonpal

**Affiliations:** 1 Internal Medicine, Brookdale University Hospital Medical Center, Brooklyn, USA; 2 Epidemiology, West Virginia University, Morgantown, USA; 3 Obstetrics and Gynecology, Maimonides Medical Center, Brooklyn, USA; 4 Gastroenterology and Hepatology, Brookdale University Hospital Medical Center, Brooklyn, USA

**Keywords:** inflammatory bowel disease, diabetes mellitus, adverse clinical outcomes, inflammatory bowel disease association with diabetes, complications of inflammatory bowel disease

## Abstract

Introduction

Inflammatory bowel disease (IBD) is increasingly common among patients with other comorbid chronic conditions, particularly diabetes mellitus (DM). Yet, studies that explored the impact of comorbid diabetes on the outcomes of IBD are scanty. Therefore, this study aims to examine the outcomes of inflammatory bowel disease among hospitalized patients with diabetes mellitus.

Methods

Using the Nationwide Inpatient Sampling (NIS) database from 2016 to 2018, we identified patients' records with a diagnosis of IBD using the International Classification of Diseases, Tenth Revision codes (ICD-10). The overall study population was further stratified by diabetes mellitus status. We matched patients with IBD and diabetes mellitus (IBD DM) with IBD cohorts using a greedy propensity score matching (PSM) ratio of 1:1 and compared in-hospital outcomes between the two cohorts. Conditional logistic regression was performed to estimate the odds of outcomes.

Results

Out of the 192,456 hospitalizations for IBD, 34,073 (7.7%) had comorbid IBD DM and 158,383 (92.3%) had no diabetes mellitus (IBD only). Patients with IBD DM are likely to be older. They have higher rates of hypertension, hyperlipidemia, coronary artery disease, obesity, peripheral vascular disease, congestive heart failure, chronic kidney disease, chronic lung disease, chronic liver disease, and stroke than the IBD cohort. After propensity score matching, IBD DM was associated with a lower adverse outcome [odds ratio (OR): 0.96, confidence interval (CI): 0.93 - 0.99, p < 0.01], IBD-related complications (intestinal or rectal fistula, intra-abdominal abscess, toxic colitis, intestinal perforation, intestinal obstruction, toxic megacolon, abscess of the abdomen, and perianal abscess), (OR: 0.76, CI: 0.72 - 0.80, P <0.01), IBD-related surgery (intestinal resections, incision, and excisions of intestine and manipulations of the rectosigmoid, rectal and perianal) (OR: 0.90, CI: 0.85 - 0.95, P <0.01). Furthermore, IBD DM was associated with a higher sepsis complication than the IBD-only cohort (OR: 1.24, CI: 1.19 - 1.30, P <0.01).

Conclusion

Our results highlight the extent to which diabetes mellitus impacts IBD outcomes and prognosis. Additionally, they emphasize the clinical awareness needed in the management of those with comorbid diseases.

## Introduction

Inflammatory bowel disease (IBD) is a chronic non-infectious inflammation of the gastrointestinal tract, associated with significant morbidities and poor quality of life [1-3]. IBD comprises Crohn's disease and ulcerative colitis, which constitute a significant clinical and public health burden affecting about 7-million people worldwide [4]. The United States contributes a quarter of the total global patients with IBD with an age-standardized prevalence rate of 464.5 per 100,000 population in 2017 [4]. IBD results from a complex interaction of the genetic and environmental factors that disrupt the immune mechanisms of the gastrointestinal tract and result in inflammation [5-6]. While IBD affects mainly the gastrointestinal tract, the associated chronic systemic inflammation leads to several extraintestinal manifestations affecting the body's major organs [7-8]. In addition, with the rise in the aging population living with IBD, there is an increased likelihood of being affected with other comorbid conditions, including cardiovascular disease or diabetes mellitus (DM) [9]. These concomitant chronic diseases modify the progression of IBD and might lead to long periods of intermittent relapses and exacerbations [10].

DM is one of the most common comorbid conditions in the United States. DM is a chronic metabolic disorder characterized by persistent impaired blood glucose metabolism [11]. According to the National Diabetes Statistics Report, 34.2-million people currently live with diabetes in the United States [12]. As a chronic disease that affects most body organs, DM can modify disease outcomes and lead to other complications. Published studies have reported IBD DM to have a higher incidence of complications, including exacerbations requiring prolonged hospitalizations and the need for surgical interventions [10]. Also, prior studies have reported DM as a covariate associated with increased risk of hospitalizations, infectious complications, colorectal cancers, and mortality [13- 14]. However, studies that describe the outcomes of IBD among diabetic patients are minimal. Understanding the hospital outcomes of comorbid IBD DM is vital for improving management and reducing morbidity and mortality. Therefore, this study sought to investigate the role of DM on IBD using a national hospital database.

## Materials and methods

Study data

This study utilized data from the Nationwide Inpatient Sample (NIS) 2016-2018 to perform a retrospective cohort study. The NIS is the largest hospital database in the United States, containing discharge records of about 8-million hospital stays annually. The NIS is a stratified, clustered database that samples discharge records from 20 percent of non-federal community hospitals. Each discharge record contains diagnoses and procedures coded using the International Classification of Disease, 10th revision (ICD-10). Institutional Review Board approval was not required for this study since the data has been de-identified.

Study population

The study population consisted of adults aged 18 and older with a diagnosis of IBD from January 1, 2016, to December 31, 2018, identified using the ICD 10: K50-K51. After excluding patients with missing age, mortality, and sex variables, we categorized the total study population into two groups: patients with IBD DM and those with IBD only. We defined DM status using ICD-10. Comparative analyses were conducted between the two groups regarding demographics such as age, sex (male and female), race/ethnicity (Whites, Blacks, Hispanics, and other races), income status (categorized into four according to the average household income of the zip-code), hospital bed size (small, medium, and large hospitals), and comorbidities (hypertension, hyperlipidemia, coronary artery disease, obesity, peripheral vascular disease, chronic heart failure, chronic kidney disease, chronic lung disease, chronic liver disease, stroke).

Study outcomes

The outcomes of this study were in-hospital adverse events, a composite of in-hospital mortality, IBD-related complications (development of fistula, abscess, colitis, perforation, intestinal obstruction, toxic megacolon), intestinal surgery (intestinal resections, incision, and excisions of intestine and manipulations of the rectosigmoid, rectal and perianal), sepsis, and septicemia, clostridium difficile infections, colorectal cancer, and resources utilization measures (length of stay and cost of hospitalizations).

Statistical analysis

Weighted values are generated to obtain a nationally representative estimate of the hospitalized patients and then produce median values and percentages for the variables. Continuous variables were expressed as weighted median values with interquartile range and compared between the cohorts using independent t-tests. In contrast, categorical variables were expressed as percentages and compared using the chi-square test. Patient demographics, comorbidities, hospital characteristics, and in-hospital outcomes were compared between males and females. We used the cost-to-charge ratio files provided by the Healthcare Cost and Utilization Project (HCUP) to convert the hospital charges to more accurate hospital costs for cost calculation. A p-value of < 0.05 was considered statistically significant.

A propensity matching method was implemented to derive two cohorts of matched samples to control potential confounding factors. A propensity score was derived for each observation via a multivariate logistic regression that models the odds of DM and the baseline characteristics. A nearest-neighbor with a ratio of 1:1, balanced propensity matching was made using a caliper width cut-off <0.2 of the standard deviation of the propensity score. A p-value of < 0.05 was considered statistically significant. After outcomes were compared between propensity score-matched subjects (IBD subjects with and without DM). The paired t-test was used to compare continuous variables and the McNemar test for categorical variables between the matched cohorts.

Finally, we developed many conditional generalized logistic regression models accounting for the matching pair, with diabetes as a primary predictor. Each of the outcomes is the dependent variable in the models. The negative binomial model was used for continuous outcomes for the length of stay and gamma distributions mode for cost. Data manipulation and statistical analyses were performed using SAS 9.4 software (SAS Institute Inc., Cary, NC). Propensity score matching (PSM) was performed using the Matchit package in R statistical software (version 3.5; R Foundation for Statistical Computing, Vienna, Austria).

## Results

Baseline characteristics of the study population

**Figure 1:**
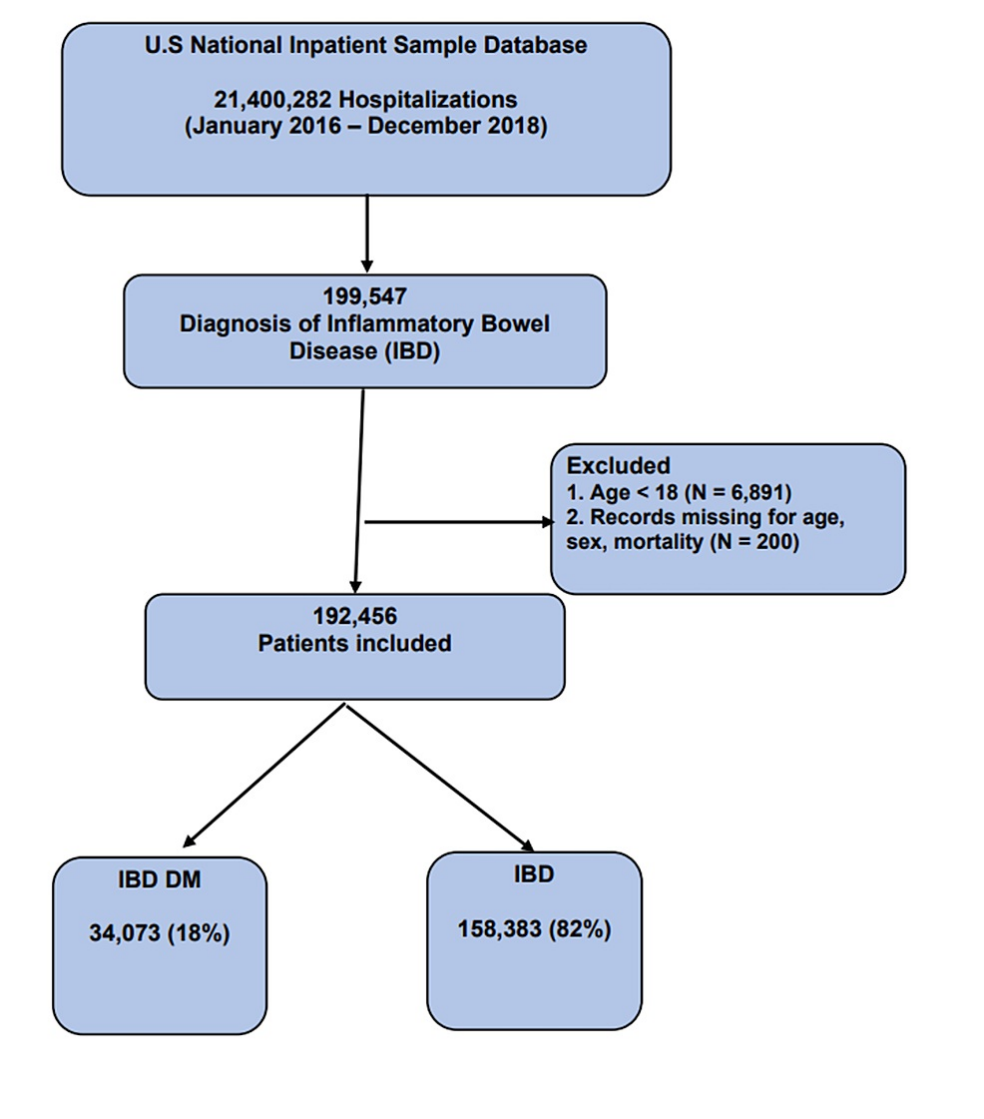
Baseline characteristics of the study population

From January 1, 2016, until December 31, 2018, a total of 21,400,282 hospitalizations were present in the NIS database. Of these hospitalizations, 192,456 had a diagnosis of IBD. DM was present in 7.7% (34,073 records), as seen in Figure 1.

There were significant differences in the baseline characteristics among the IBD DM and IBD cohorts. Compared to IBD patients, the IBD DM cohorts were older (median age 64.6 vs. 50.8), female (53.6% vs. 46.4%), and had a higher percentage of comorbidities than the IBD-only cohort. The baseline patient and hospital characteristics of the two groups are shown in Tables 1-2.

**Table 1:** Baseline characteristics of patients with inflammatory bowel disease and diabetes mellitus (Nationwide Inpatient Sampling database 2016 -2018) Values are expressed in percentages unless otherwise stated. WE: weighted estimates; IBD: inflammatory bowel disease; DM: diabetes mellitus; IQR: interquartile range

Variables	Total	IBD DM	IBD	p-value
	192,456	(N = 34,073)	(N =158,383)	
	(WE: 962,280)	(N = 170,365)	(N = 791,915)	
Age (median, IQR)	54.0 (36.7 - 68.5)	64.6 (53.7 - 73.5)	50.8 (34.4 - 66.5)	<0.0001
Age category				
18 -34	20.7	3.8	24.3	<0.0001
35 - 54	29.3	21.7	30.9	
55 - 74	34.2	50.9	30.6	
≥ 75	15.8	23.6	14.1	
Sex				
Male	43.7	46.4	43.1	<0.0001
Female	56.3	53.6	56.9	
Race				
White	76.0	74.7	76.3	<0.0001
Black	10.9	12.0	10.6	
Hispanic	6.0	6.4	5.9	
Other	3.7	3.9	3.7	
Unknown	3.4	3.0	3.5	
Comorbidities				
Hypertension	38.2	63.2	32.9	<0.0001
Hyperlipidemia	22.8	47.6	17.4	<0.0001
Coronary Artery Disease	12.7	27.7	9.5	<0.0001
Obesity	12.5	24.8	9.8	<0.0001
Peripheral Vascular Disease	4.9	7.7	4.3	<0.0001
Chronic Heart Failure	9.8	21.4	7.3	<0.0001
Chronic Kidney Disease	6.5	14.2	4.8	<0.0001
Chronic Lung Disease	20.9	29.8	19.0	<0.0001
Chronic Liver Disease	6.8	10.1	6.1	<0.0001
Stroke	5.8	10.2	4.9	<0.0001
Smoking	16.9	13.5	17.6	<0.0001
Hospital Bed Size				
Small	19.1	19.6	19.0	<0.0001
Medium	28.1	29.4	27.8	
Large	52.9	51.0	53.3	
Median Household Income				
< 25th percentile	27.2	27.2	23.8	<0.0001
26-50th percentile	27.2	27.2	25.3	
51-75th percentile	24.8	24.8	26.3	
76-100th percentile	20.8	20.8	24.6	

**Table 2:** Baseline characteristics of patients with inflammatory bowel disease and diabetes mellitus, after propensity matching Values are expressed in percentages unless otherwise stated. SMD: standardized mean differences; IBD: inflammatory bowel disease; DM: diabetes mellitus; IQR: interquartile range

Variables	IBD DM	IBD	SMD (%)
Observations	(N = 33,870)	(N = 33,870)	
Age (median, IQR)	64.5 (53.7 - 73.4)	66.4 (54.6 - 76.0)	10.3
Sex			3.0
Male	46.3	46.2	
Female	53.7	53.8	
Race			3.9
White	74.7	78.7	
Black	12.0	9.2	
Hispanic	6.4	4.8	
Other	3.9	3.3	
Unknown	3.0	4.0	
Comorbidities			
Hypertension	63.0	65.6	5.5
Hyperlipidemia	47.3	46.6	1.5
Coronary Artery Disease	27.4	26.1	3.0
Obesity	24.5	21.9	6.1
Peripheral Vascular Disease	7.7	7.8	0.3
Chronic Heart Failure	21.1	19.2	4.7
Chronic Kidney Disease	14.0	12.3	4.9
Chronic Lung Disease	29.7	30.2	1.1
Chronic Liver Disease	10.1	9.8	1.0
Stroke	10.2	10.1	0.4
Smoking	13.6	13.7	0.3
Hospital Bed Size			3.0
Small	19.6	19.6	
Medium	29.4	29.2	
Large	51.0	51.2	
Median Household Income			1.2
< 25th percentile	27.1	26.5	
26-50th percentile	27.2	27.0	
51-75th percentile	24.9	25.6	
76-100th percentile	20.8	20.8	

In-hospital outcomes are depicted in Table 3. Overall, the IBD DM cohorts had a lower prevalence of major in-hospital adverse events than the IBD cohorts. Compared to the IBD group, the IBD DM had lower IBD-related complications (9.3% vs. 14.5%, p < 0.01), intestinal surgeries (26.0% vs. 38.5%, p < 0.01). On the other hand, the IBD DM group had a higher prevalence of sepsis and septicemia complications (14.7% vs 10.8%, p < 0.01) and in-hospital mortality (2.2% vs 1.4%, p < 0.01). The prevalence of clostridium difficile infections and colorectal cancer are similar between the two groups. Similarly, the length of hospital stay and cost- were more prevalent among the IBD DM group than the IBD only group.

**Table 3:** In-hospital outcomes of inflammatory bowel disease among IBD DM vs IBD patients before propensity score matching IBD: inflammatory bowel disease; DM: diabetes mellitus; IQR: interquartile range

In-hospital Outcomes	Total	IBD DM	IBD	p-value
Major Adverse events	40.6%	36.1%	41.6.1%	<0.0001
IBD-related complications	13.5%	9.3%	14.5%	<0.0001
Surgery	36.3%	26.0%	38.5%	<0.0001
Sepsis/Septicemia	11.5%	14.7%	10.8%	<0.0001
C.difficile infections	4.6%	4.7%	4.6%	0.4205
Colorectal Cancer	0.9%	1.0%	0.9%	0.9339
Death	1.5%	2.2%	1.4%	<0.0001
Length of stay (Median (IQR)	3.1(1.7 - 3.7)	3.5(1.9 - 6.3)	3.0(1.7 - 5.5)	<0.0001
Cost (Median (IQR)	8,224 (4,986 - 14,761)	9,224 (5,582 - 16,212)	8,036 (4,873 - 14446)	<0.0001

**Figure 2:**
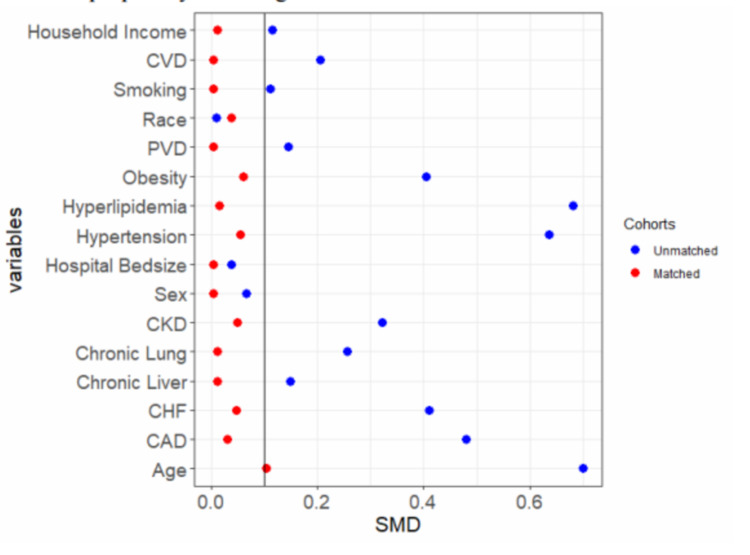
Dot plots showing the standardized mean differences of the baseline variables before and after propensity matching CVD: cerebrovascular disease; PVD: peripheral vascular disease; CKD: chronic kidney disease; CHF: congestive heart failure; CAD: coronary artery disease

**Figure 3:**
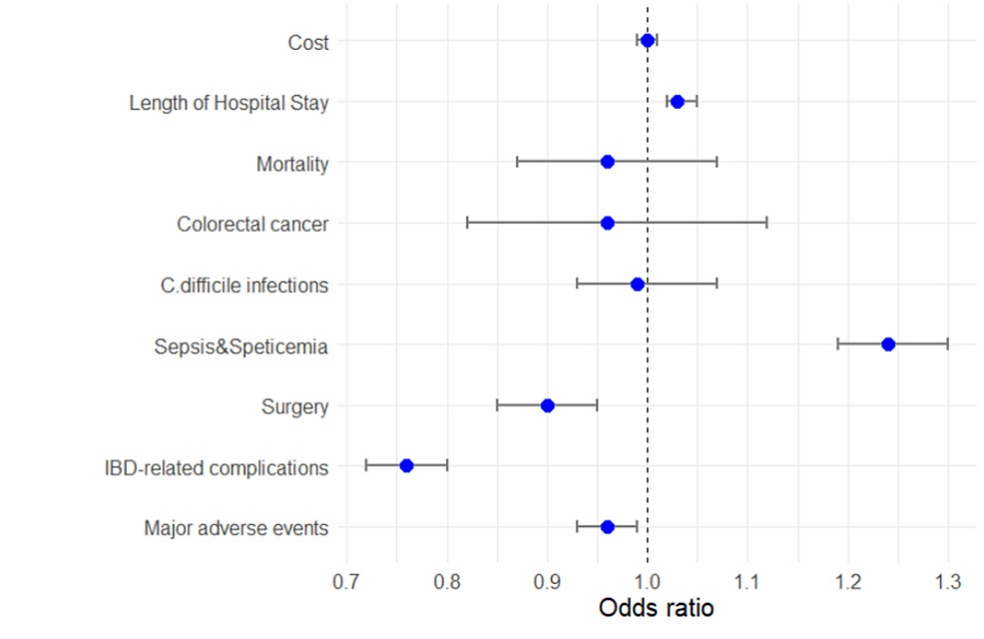
Plots of the adjusted odds ratio of clinical outcomes of inflammatory bowel disease and diabetes mellitus after propensity matching

**Figure 4:**
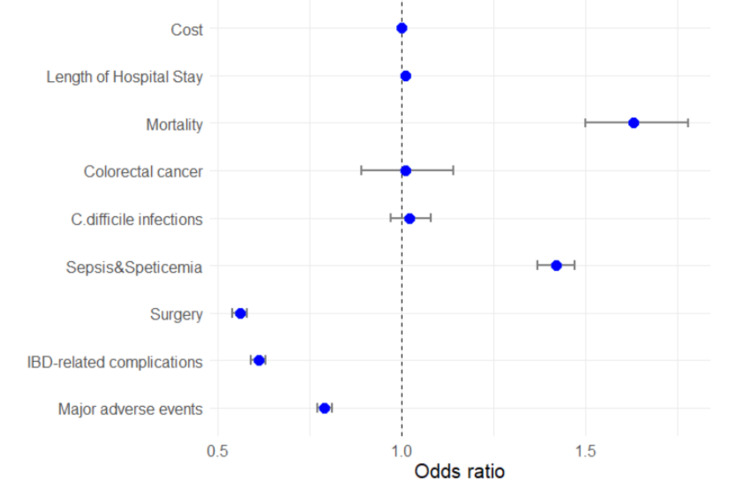
Plot of the unadjusted odds ratio of clinical outcomes of inflammatory bowel diseases and diabetes mellitus

Using propensity matching, we adjusted for baseline demographics and hospital characteristics to generate matched cohorts of IBD DM and IBD groups (n = 33,870) (Table 4). We achieved the variables' standardized mean differences between the two groups to arrive at a well-matched cohort. The overall in-hospital adverse events is still lower among the IBD DM cohort as compared to the IBD group (36.2% vs. 37.2%, OR: 0.96, CI: 0.93 - 0.99, p < 0.01). Furthermore, the IBD DM cohort had a lower incidence of IBD-related complication (9.3% vs. 11.9%, OR: 0.76, CI: 0.72 - 0.80, P <0.01) and surgery (26.0% vs. 28.9%, OR: 0.90, CI: 0.85 - 0.95, P <.01). However, the DM group had a higher sepsis complication compared to the IBD group (14.7% vs. 12.2%, OR: 1.24, CI: 1.19 - 1.30, P <0.01). See Figures 2-4 and Table 5.

**Table 4:** In-hospital outcomes of inflammatory bowel disease among IBD DM vs IBD patients after propensity score matching IBD: inflammatory bowel disease; DM: diabetes mellitus; IQR: interquartile range

In-hospital Outcomes	IBD DM	IBD	OR (95% CI)	p-value
Major Adverse events	36.2	37.2	0.96 (0.93 - 0.99)	0.0046
IBD-related complications	9.3	11.9	0.76 (0.72 - 0.80)	<0.0001
Surgery	26.0	28.9	0.90 (0.85 - 0.95)	0.0004
Sepsis/Septicemia	14.7	12.2	1.24 (1.19 - 1.30)	<0.0001
C.difficile infections	4.7	4.7	0.99 (0.93 - 1.07)	0.8567
Colorectal Cancer	1.0	1.0	0.96 (0.82 - 1.12)	0.5841
Death	2.2	2.3	0.96 (0.87 - 1.07)	0.4670
Length of stay (Median IQR)	3.5 (1.9 - 6.3)	3.3 (1.8 - 6.1)	1.03 (1.02 - 1.05)	<0.0001
Cost (Median IQR)	9,216 (5,578 - 16,199)	9,147 (5,471 - 16,272)	1.00 (0.99 - 1.01)	0.8839

**Table 5:** Baseline characteristics of patients with inflammatory bowel disease and diabetes mellitus, after propensity matching Values are expressed in percentages unless otherwise stated. SMD: standardized mean differences; IBD: inflammatory bowel disease; DM: diabetes mellitus; IQR: interquartile range

Variables	IBD DM	IBD	SMD (%)
Observations	(N = 33,870)	(N = 33,870)	
Age (median, IQR)	64.5 (53.7 - 73.4)	66.4 (54.6 - 76.0)	10.3
Sex			3.0
Male	46.3	46.2	
Female	53.7	53.8	
Race			3.9
White	74.7	78.7	
Black	12.0	9.2	
Hispanic	6.4	4.8	
Other	3.9	3.3	
Unknown	3.0	4.0	
Comorbidities			
Hypertension	63.0	65.6	5.5
Hyperlipidemia	47.3	46.6	1.5
Coronary Artery Disease	27.4	26.1	3.0
Obesity	24.5	21.9	6.1
Peripheral Vascular Disease	7.7	7.8	0.3
Chronic Heart Failure	21.1	19.2	4.7
Chronic Kidney Disease	14.0	12.3	4.9
Chronic Lung Disease	29.7	30.2	1.1
Chronic Liver Disease	10.1	9.8	1.0
Stroke	10.2	10.1	0.4
Smoking	13.6	13.7	0.3
Hospital Bed Size			3.0
Small	19.6	19.6	
Medium	29.4	29.2	
Large	51.0	51.2	
Median Household Income			1.2
< 25th percentile	27.1	26.5	
26-50th percentile	27.2	27.0	
51-75th percentile	24.9	25.6	
76-100th percentile	20.8	20.8	

## Discussion

In this study, we examined the in-hospital outcomes of IBD among patients with DM. In addition, we compared IBD patients with comorbid DM to those without diabetes using a propensity score matching method. The main findings of our study are as follows: (1) IBD DM patients have a significant increase in the odds of developing sepsis and septicemia compared to the IBD only cohort ; (2) IBD DM is associated with a decrease in the odds of having IBD-related complications and surgical interventions compared to IBD cohorts; (3) there were no significant differences in the risk of mortality, clostridium difficile infections, or colorectal cancer, among IBD DM and IBD.

To date, only a few clinical or epidemiological studies had investigated the IBD outcomes among diabetic patients. A recent longitudinal cohort study conducted in the US found that comorbid IBD DM patients have a significantly increased risk of IBD-related hospitalization, complications, surgical interventions, and all-cause mortality [10]. Compared with IBD cohorts, IBD DM patients had significantly higher rates of sepsis and other infections [10]. Furthermore, a retrospective study that utilized 2810 outpatient cohorts concluded that DM is associated with worse IBD severity reflected by increased use of the emergency room and nearly double the rates of patients with gastrointestinal clinic visits [15]. While these studies have employed different databases and methodology, one consistent finding with these studies is the overall increase in the odds of infection-related complications among IBD DM patients compared to the IBD cohorts. This was consistent with our study that found an increase in the odds of having sepsis and septicemia among IBD DM patients.

Several mechanisms might explain the reason for the increased incidence of infectious complications among the IBD DM cohort. IBD and DM are both autoimmune disease conditions that disrupt the entire immune system [16-17]. These disease states are associated with dysregulation of the intestinal immune barrier, promoting local and systemic inflammation, explaining the increased susceptibility to several infections [18-19]. The dysregulation also leads to the release of multiple cytokines, including tumor necrosis factor (TNF)-alpha and interleukins [20-21]. The inhibited secretions of the interleukins in diabetic patients cause a defect in the antigen-presenting cells, monocytes, and contribute to reduced immunity [22]. Furthermore, the hyperglycemic state in DM affects the complement system and contributes to reduced neutrophil function [23]. Also, IBD patients are usually on immunosuppressive medications, which reduces the function of the overall immune system and predisposes them to an increased risk of opportunistic infections [24].

Given the overlapping impairment in the immune system, concomitant DM is a significant comorbidity among patients with IBD, as reflected by increased hospitalizations [15]. While our study did not find a significant difference in the length of hospital stay and cost of hospitalization between IBD DM and IBD, we did not explore health care utilization. A study found that IBD DM patients had a higher outpatient prescription and antibiotic usage than IBD cohorts [15]. Also, IBD DM patients' increased emergency department and gastrointestinal clinic visits reflected the important burden of IBD DM comorbidity [15].

The use of propensity score matching (PSM) is one of the strengths of this study. The PSM methodology has been compared to the randomized clinical trials, as it effectively adjusts for confounders and produces estimates close to those derived from randomized clinical trials [25-26]. PSM facilitates comparability between the IBD DM and IBD cohorts, making it a valuable technique for assessing the risk factors between these groups. Therefore, PSM minimizes several biases and limitations of a large observational study like ours. The additional strengths of our study include utilizing an extensive, nationwide inpatient database, which makes the results generalizable to the entire population. Despite these strengths, our findings still needed to be interpreted in light of some limitations. The comorbidities in the nationwide inpatient sample database rely heavily on using the diagnostic coding ICD-10. Any error in coding can affect the study's validity. Thus, there might be variations, which is also a limitation that could affect the results generated. The PSM methodology was designed to minimize confounding that could bias the results from observed covariates. However, PSM cannot address unobserved factors, which may still lead to biased results. Also, our study did not explore the different outcomes regarding the variants of IBD. IBD, comprising Crohn's disease and ulcerative colitis, can have different results. The retrospective nature of this study is a limitation and the diagnosis of diabetes mellitus based on ICD-10 codes alone might be unreliable, thus some of the patients in the IBD-only group might also have diabetes mellitus but are yet to be diagnosed, which can affect the results of this study. A prospective study will be helpful to better study the complications of inflammatory bowel disease in patients with diabetes mellitus.

## Conclusions

This study shows that DM is a determinant of severe disease and increased risk of severe infectious complications in hospitalized diabetic patients with IBD. The use of PSM is one of the strengths of this study, as it effectively adjusts for confounders and minimizes several biases and limitations of a large observational study. More longitudinal and prospective studies are required to further study the impact of diabetes among patients with IBD.
